# Influence of hypoxia and irradiation on osteopontin expression in head and neck cancer and glioblastoma cell lines

**DOI:** 10.1186/s13014-015-0473-x

**Published:** 2015-08-12

**Authors:** Gisela Wohlleben, Agmal Scherzad, Antje Güttler, Dirk Vordermark, Sebastian Kuger, Michael Flentje, Buelent Polat

**Affiliations:** Department of Radiation Oncology, University hospital Wuerzburg, Josef-Schneider-Straße 11, 97080 Würzburg, Germany; Department of Oto-Rhino-Laryngology, Plastic, Aesthetic and Reconstructive Head and Neck Surgery, University hospital Wuerzburg, Wuerzburg, Germany; Department of Radiotherapy, Martin-Luther-University Halle-Wittenberg, Halle, Saale Germany; Research Unit of Radiation Cytogenetics, Helmholtz–Zentrum München, German Research Center for Environmental Health, Neuherberg, Germany

**Keywords:** Osteopontin, Hypoxia, Irradiation, Head and neck cancer, Glioblastoma multiforme

## Abstract

**Background:**

Tumor hypoxia is a known risk factor for reduced response to radiotherapy. The evaluation of noninvasive methods for the detection of hypoxia is therefore of interest. Osteopontin (OPN) has been discussed as an endogenous hypoxia biomarker. It is overexpressed in many cancers and is involved in tumor progression and metastasis.

**Methods:**

To examine the influence of hypoxia and irradiation on osteopontin expression we used different cell lines (head and neck cancer (Cal27 and FaDu) and glioblastoma multiforme (U251 and U87)). Cells were treated with hypoxia for 24 h and were then irradiated with doses of 2 and 8 Gy. Osteopontin expression was analyzed on mRNA level by quantitative real-time RT-PCR (qPCR) and on protein level by western blot. Cell culture supernatants were evaluated for secreted OPN by ELISA.

**Results:**

Hypoxia caused an increase in osteopontin protein expression in all cell lines. In Cal27 a corresponding increase in OPN mRNA expression was observed. In contrast the other cell lines showed a reduced mRNA expression under hypoxic conditions. After irradiation OPN mRNA expression raised slightly in FaDu and U87 cells while it was reduced in U251 and stable in Cal27 cells under normoxia. The combined treatment (hypoxia and irradiation) led to a slight increase of OPN mRNA after 2 Gy in U251 (24 h) and in U87 (24 and 48 h) cell lines falling back to base line after 8 Gy. This effect was not seen in Cal27 or in FaDu cells. Secreted OPN was detected only in the two glioblastoma cell lines with reduced protein levels under hypoxic conditions. Again the combined treatment resulted in a minor increase in OPN secretion 48 hours after irradiation with 8 Gy.

**Conclusion:**

Osteopontin expression is strongly modulated by hypoxia and only to a minor extent by irradiation. Intracellular OPN homeostasis seems to vary considerably between cell lines. This may explain the partly conflicting results concerning response prediction and prognosis in the clinical setting.

## Background

Tumor hypoxia is a known risk factor for reduced response to radiotherapy [1]. In these studies hypoxia was measured with invasive polarographic O_2_ needle electrodes. The evaluation of noninvasive methods for the detection of hypoxia is of interest. One strategy is the use of imaging modalities like PET/CT or MRI. Common PET tracers for hypoxia imaging which were used in clinical trials are 18-F-MISO, 18-F-FAZA and 62-Cu ATSM [2–4].

Another way to investigate tumor hypoxia is the search for hypoxia associated biomarkers which can be detected from blood samples or tumor tissues. Several studies investigated the role of HIF-1α and its dependent gene regulation including CA IX and VEGF [1, 5–7]. One of these so called endogenous hypoxia markers is osteopontin (OPN) [8], a secreted glycophosphoprotein which is overexpressed in many cancer types [9]. It plays an important role in tumor progression, angiogenesis and metastasis formation [10]. Furthermore, OPN is a prognostic marker for different tumor entities in which elevated plasma levels are associated with an unfavorable prognosis [11–15]. In head and neck cancer treated with definitive radiotherapy Petrik et al. demonstrated prolonged overall survival and event free survival in patients with OPN levels below median [11]. Similar results were shown by Overgaard et al. and Snitcovsky et al. [13, 16]. However, the TROG 02.02 phase III trial did not report any correlation between osteopontin levels and tumor control or patient outcome [17].

To look at possible intervening variables we analyzed the influence of irradiation on OPN expression under normoxic and hypoxic conditions in head and neck cancer and glioblastoma cell lines.

## Methods

### Cell culture

The human tumour cell lines Cal 27 (tongue carcinoma), FaDu (hypopharyngeal carcinoma), U251 and U87 (both derived from glioblastoma multiforme) were purchased from the American Type Culture Collection (ATCC, Manassas, VA, USA). Cells were grown in Dulbecco’s modified Eagle`s medium (DMEM) supplemented with 10 % fetal bovine serum (PAA, Cölbe Germany), 2 mM L-glutamine and penicillin (100 IU/ml)/streptomycin (100 μg/ml) under normoxic standard conditions (21 % O_2_, 5 % CO_2_ at 37 °C).

To compare OPN expression under normoxic and hypoxic standard conditions at mRNA or protein levels cells were seeded on Petri dishes at a concentration of 8×10^5^/dish (Cal 27) or 1×10^6^/dish (FaDu, U87, U251). Standard condition for hypoxic treatment of cells is normally at 0.1 % oxygen concentration in radiobiology. Afterwards cells were allowed to adhere before part of them were transferred into a hypoxic glove box (Invivo_2_ 200, Ruskinn technology limited, Leeds, UK) and cultured at 0.1 % O_2_ (5 % CO_2_ at 37 °C; FaDu, U87, U251) or 1.0 % (5 % CO_2_ at 37 °C; Cal 27, FaDu) for 24 hours. Since the Cal27 cells did not survive under 0.1 % O_2_ concentration for more than 24 hours (which is the incubation time required before irradiation) we conducted the experiments under less hypoxic conditions with 1 % O_2_. For a better comparability of the hypoxic treatment in the head and neck cancer cell lines we repeated the western blot and qPCR analysis in the FaDu cell line with 1 % O_2_. In parallel cells were kept under normoxic conditions. Twenty-four hours later cells were irradiated at doses of 2 Gy and 8 Gy (still under hypoxic or normoxic conditions) using a 6MV linear accelerator (Siemens, Concord, CA, USA) at a dose rate of 2 Gy/min or left untreated. After 24 or 48 hours post irradiation growing under standard or hypoxic conditions respectively, cells were harvested for preparing whole-cell lysates or RNA.

### Western blot analysis

Whole-cell lysates were prepared by lysing cells in RIPA buffer under standard conditions. Proteins were run on 4-12 % Bis-Tris gradient gels (Invitrogen, Karlsruhe, Germany), electrophoretically transferred to polyacrylamide membranes (Invitrogen) and analyzed by Western blotting. Polyclonal rabbit anti-human-OPN antibody FL-314 and monoclonal mouse anti-human-HPRT antibody were obtained from Santa Cruz Biotechnology, Inc. (Heidelberg, Germany). Mouse monoclonal antibodies against β-actin and GAPDH were purchased from Sigma (Deisenhofen, Germany) and abcam (Cambridge, UK). Detection was achieved using species-specific horseradish peroxidase-coupled secondary antibodies (DAKO, Hamburg, Germany) and Amersham™ ECL™ Select Western blotting detection reagent (GE Healthcare, Chalfont St. Giles, Buckinghamshire, UK).

### ELISA for osteopontin detection in supernatants

Osteopontin protein levels in the non-concentrated supernatants of cultured cells, collected after indicated durations of hypoxia, with or without irradiation, were determined by ELISA, using the Human Osteopontin Assay Kit-IBL (Immuno-Biological Laboratories Co., Ltd.) according to the manufacturer’s instructions.

### RNA isolation and quantitative real-time PCR (qPCR)

RNA of the cells was isolated using the RNeasy® Mini Kit as recommended by the manufacturer (Qiagen, Hilden, Germany). Afterwards RNA was reverse transcribed into cDNA using First Strand cDNA Synthesis Kit (Fermentas GmbH, St. Leon-Rot, Germany) following the instructions given by the suppliers. To amplify cDNA coding for OPN and the housekeeping gene hypoxanthine phosphoribosyltransferase (HPRT), quantitative real-time-PCR was carried out using TaqMan® Gene Expression Assays (Applied Biosystems, Foster City, CA, USA). To verify that the hypoxic culture conditions were sufficient CA IX qPCR analysis was performed in the same way. The cycle number crossing the threshold was used as the threshold cycle (C_t_). The amount of cDNA in each sample was normalized to the crossing point of the housekeeping gene hypoxanthine phosphoribosyltransferase (HPRT). Changes in the expression were detected and quantified using the comparative CT method (2^−ΔΔCt^).

### Statistics

All experiments were carried out in triplicate and reproduced 2 times. Data are presented as means (± standard deviation). Statistical analysis was performed using ANOVA between groups. Significance level was set at p < 0.05.

## Results

### Effect of hypoxia and irradiation on OPN expression - protein level

**Fig. 1:**
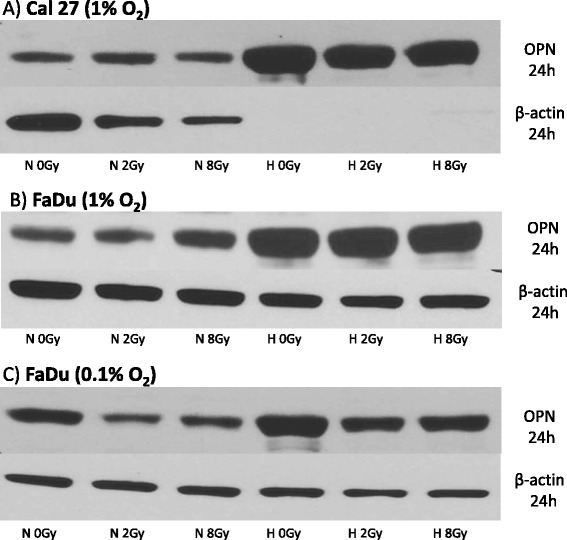
Western blot showing OPN protein expression under normoxic (N = 21 % O_2_) and hypoxic (H = 1 % O_2_ or 0.1 % O_2_) conditions 24 h after irradiation with 0, 2 and 8 Gy in Cal27 (**a**) and FaDu (**b**), (**c**) head and neck cancer cell lines. Hypoxic conditions were 1 % O_2_ for both cell lines. FaDu cells were also treated with 0.1 % O_2_ (**c**) showing comparable results

**Fig. 2:**
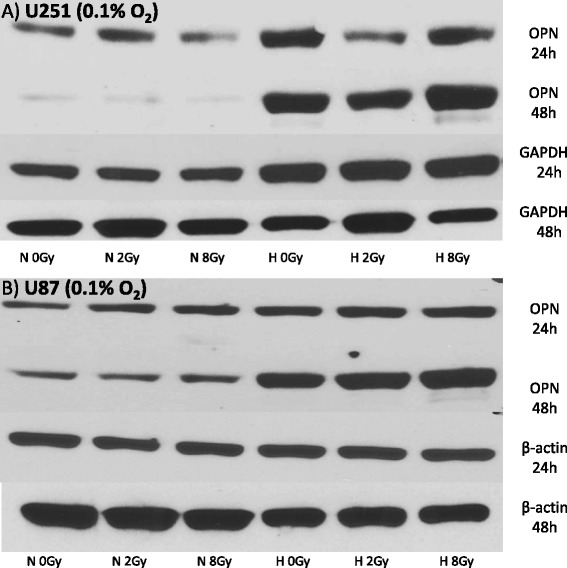
Western blot showing OPN-expression under normoxic (N = 21 % O_2_) and hypoxic (H = 0.1 % O_2_) conditions 24 h and 48 h after irradiation with 0, 2 and 8 Gy in U251 (**a**) and U87 (**b**) glioblastoma cell lines

Two different head and neck cancer cell lines (tongue carcinoma cell line, CAL 27 and hypopharyngeal cancer cell line FaDu) and two glioblastoma cell lines (U251 and U87) were cultured under different oxygen conditions (1 % O_2_ for Cal27 and FaDU and 0.1 % O_2_ for U251 and U87). Furthermore, a possible effect on OPN expression caused by irradiation (2 Gy or 8 Gy) was investigated. Twenty-four hours (all cell lines) and 48 hours (U251, U87) after irradiation, lysates of the different cell lines were prepared and tested by Western blot analysis for OPN protein levels. Twenty-four hours after irradiation a clear increase of OPN protein expression was seen in all cell lines grown under hypoxic conditions compared with normoxic controls. Except for FaDu and U251 cells grown at 0.1 % O_2_, this effect was independent of additional irradiation (Figs. 1 and 2). Irradiation of FaDu with doses of 2 and 8 Gy apparently had a weak inhibitory effect of OPN levels under hypoxic standard culture conditions (0.1 % O_2_). This was also seen in U251 cells under hypoxia and 24 h post irradiation with 2 Gy. U251 and U87 cells, which survived 48 hours post irradiation under hypoxic conditions, showed a significant increase of OPN expression 48 hours after irradiation compared with cells in the normoxic control group (Fig. 2). Interestingly, in both glioblastoma cell lines a trend for a reduced osteopontin content was seen under normoxic conditions after 48 hours irrespective of irradiation (Fig. 2). Beta-actin and GAPDH were used as loading controls, respectively. The missing protein bands of ß-actin (and GAPDH, data not shown) in lysates of Cal 27 cells, grown under hypoxic conditions, can be explained by the altered expression of housekeeping genes under hypoxia in some cell lines [18, 19].

### Effect of hypoxia and irradiation on OPN expression – mRNA level

**Fig. 3:**
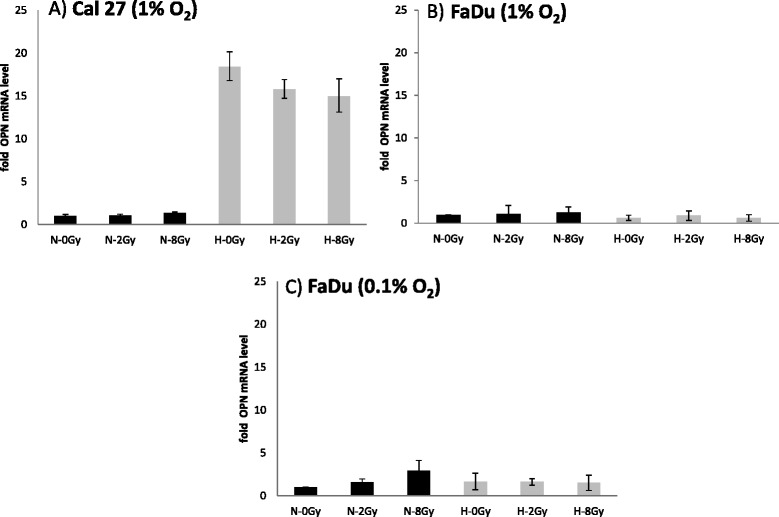
Quantitative real-time RT-PCR data showing OPN RNA expression under normoxic (N = 21 % O_2_) and hypoxic (H = 1 % O_2_ or 0.1 % O_2_) conditions 24 h after irradiation with 0, 2 and 8 Gy in Cal27 (**a**) and FaDu (**b**), (**c**) head and neck cancer cell lines

To assess hypoxia induced effects alone or in combination with irradiation (2 Gy or 8 Gy) on OPN mRNA levels qPCR was performed. Cal 27 cells showed a trend towards increased OPN mRNA levels (up to 12 fold, p = 0.2) under hypoxic conditions, which was slightly reduced by irradiation. In FaDu cells hypoxia (0.1 % O_2_) caused an insignificant increase in OPN mRNA levels, which was hardly influenced by irradiation. Since the Cal27 cells did not survive under 0.1 % O_2_ concentration for more than 24 hours (the time point of irradiation) we conducted the experiments under less hypoxic conditions with 1 % O_2_. Otherwise irradiation and growing the cells 24 h post irradiation would not be possible. For a better comparability of the hypoxic treatment we repeated the western blot and qPCR analysis in the FaDu cell line with 1 % O_2_. We observed comparable results in osteopontin expression whether the cells were treated with 0.1 % or 1 % O_2_ in the FaDu cell line (Fig. 3).

**Fig. 4:**
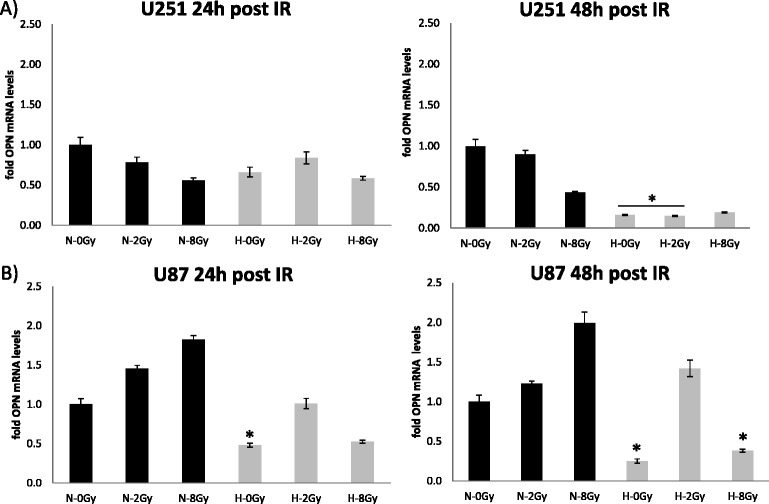
Quantitative real-time RT-PCR data showing OPN RNA expression under normoxic (N = 21 % O_2_) and hypoxic (H = 0.1 % O_2_) conditions 24 h and 48 h after irradiation (IR) with 0, 2 and 8 Gy in U251 (**a**) and U87 (**b**) glioblastoma cell lines. *marks statistical differences between normoxic and hypoxic conditions on the corresponding dose levels (ANOVA; p < 0.05)

In contrast there was a decrease in OPN mRNA levels in the glioblastoma cell lines when cells were cultured under standard hypoxia (see Fig. 4: 24 h post irradiation), which was more pronounced the longer the cells were in culture (see Fig. 4: 48 h post irradiation) (p-values < 0.05). Doses of 2 Gy led to a non-significant increase of OPN mRNA expression under hypoxic conditions (Fig. 4).

To confirm hypoxic culture conditions, quantitative real-time PCR for CA IX expression (which is HIF-1α dependent and up-regulated under hypoxia) was performed. FaDu and U251 cells showed a clear increase (30–60 fold) of CA IX expression under hypoxic conditions. Cal27 and U87 cells reacted to hypoxia with a 2-fold and 5-fold increase, respectively (data not shown). Changes in CA IX expression under hypoxia were statistically significant in all cell lines.

### Effect of hypoxia and irradiation on OPN secretion into cell culture medium

**Fig. 5:**
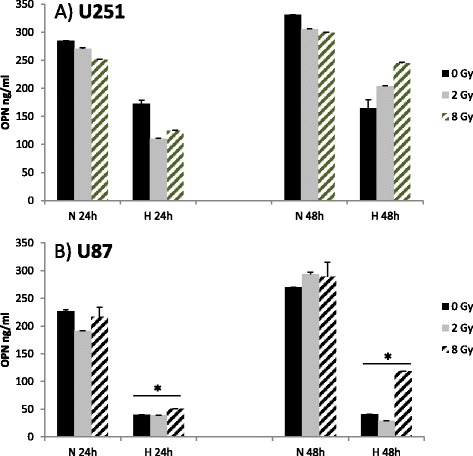
ELISA from cell culture supernatants showing OPN-concentrations under normoxic (N = 21 % O_2_) and hypoxic (H = 0.1%O_2_) conditions 24 h and 48 h after irradiation with 0, 2 and 8 Gy in U251 (**a**) and U87 (**b**) glioblastoma cell lines. *marks significant differences between normoxic and hypoxic conditions with p < 0.05 for 24 and 48 hours

For detection of osteopontin we used a commercial ELISA system without further preparation (e.g. concentration) of the cell supernatants. There was no secreted osteopontin detectable in the supernatants of the two head and neck cancer cell lines Cal27 and FaDu (data not shown). U251 and U87 cells were actively secreting osteopontin which was reduced under hypoxic conditions at 24 h and 48 h (means time after irradiation). In U87 cells this reduction reached statistical significance (p < 0.05). In the same cell line there was a slight increase in OPN concentrations after irradiation with 8 Gy after 48 hours compared to 0 and 2 Gy under hypoxic conditions which was not statistically significant (Fig. 5).

## Discussion

Osteopontin plays a critical role in cancer progression by binding and activating different αvβ - integrins and CD44 receptors. Via intracellular signaling this leads to an increase in cancer cell motility, enhanced proliferation, anchor independence growth and other steps necessary for metastasis formation. A recent overview of this multifunctional protein and other members of the matricellular protein family is given by Chong et al. [10]. In head and neck cancer as well as in glioblastoma patients osteopontin has been shown to have prognostic impact where high osteopontin plasma levels were associated with worse outcome [11, 13, 14]. In previous work we showed in different cancer cell lines that silencing osteopontin expression resulted in enhanced radiosensitivity [20–22]. This was also true for the U251 glioblastoma cell line where we observed an osteopontin dependent decrease in cell proliferation, migration and apoptotic activity and finally in a reduced clonogenic survival [14]. Induction of OPN after irradiation was seen by Chang et al. in A549 cells by p53 signaling which in the end led to an inhibition of the apoptotic pathway and resulted in more radio-resistant tumor cells [23].

In our experiments tumor hypoxia was associated with elevated OPN expression. In *in-vivo* trials osteopontin expression was correlated with tumor hypoxia measured invasively with the Eppendorf electrode in head and neck and lung cancer patients [24, 25]. Hence, osteopontin signaling may serve as an endogenous biomarker for tumor hypoxia and a possible intrinsic target. In a panel of standardized *in-vitro* cell lines we confirmed increased OPN protein expression during hypoxia. Irradiation itself did not change this pattern significantly when studied 24 and 48 hours after hypoxic challenge. This was also observed in a breast cancer cell line after irradiation with 2 Gy [22]. If an increase in OPN expression would have been observed this could be beneficial for tumor cell survival and may reduce treatment response. This may result in an increase in radio-resistance.

Unexpectedly, in glioblastoma cell lines a considerable decrease in osteopontin secretion (40-85 %) under hypoxic conditions with only a slight increase when cells were irradiated. A decrease in OPN transcription rates were seen in our qPCR experiments. The reduction of transcriptional activity of OPN mRNA might be an effect of a negative feed-back loop due to intracellular accumulation of osteopontin protein. This assumption is supported by the observation that intracellular OPN protein levels increase while active secretion was reduced by hypoxia. An intense literature search for OPN expression patterns under irradiation and hypoxia did not reveal a comparable paper in glioblastoma. A recent paper by our group showed an increased OPN mRNA expression in glioblastoma tumor tissue compared to adjacent non tumor tissue. This is in contrast to our findings in our cell culture model, since we experienced a decrease of OPN mRNA in the two glioblastoma cell lines [26]. To elucidate whether the decrease of OPN mRNA or the intracellular increase of OPN protein has an effect on cell proliferation, migration or clonogenic survival further experiments will be done by our group in the near future. It would also be interesting to know, if the reduced secretion of OPN can reduce functional activity of the cells in an autocrine manner. We could demonstrate in previously published articles in lung, breast and endometrial cancer cell lines that silencing osteopontin expression resulted in reduced cell proliferation, cell migration and enhanced radiosensitivity [20–22]. This was also true for the U251 glioblastoma cell line where we observed an osteopontin dependent decrease in cell proliferation, migration and apoptotic activity and finally in a reduced clonogenic survival [14].

A different expression pattern was seen in the two head and neck cancer cell lines. No secretion of osteopontin at baseline and after treatment with hypoxia or irradiation was detected in cell culture supernatants (data not shown). Likewise, Hui et al. detected OPN in supernatants in only one of four evaluated nasopharyngeal cell lines. In their hands OPN secretion was not affected by hypoxic treatment [27]. In our study higher protein levels were correlated with increasing OPN mRNA expression under hypoxia in Cal27 and to a lesser degree in FaDu cells. Hypoxia enhanced osteopontin expression in head and neck cancer cells was already demonstrated by Zhu et al. who described a hypoxia associated element in the osteopontin promotor region regulated by a Ras enhanced activator [28].

In a next step we are going to analyze intracellular signaling of osteopontin under these experimental conditions and will evaluate functional activity of the tumor cells via proliferation, migration and survival assays.

## Conclusions

In conclusion, osteopontin expression is strongly modulated by hypoxia and only to a minor extent by irradiation. Intracellular OPN homeostasis seems to vary considerably between cell lines and differ between mRNA and protein expression. This may explain the partly conflicting results concerning response prediction and prognosis in the clinical setting.
